# TMEM147 is a novel biomarker for diagnosis and prognosis of hepatocellular carcinoma

**DOI:** 10.1590/1678-4685-GMB-2022-0323

**Published:** 2023-06-16

**Authors:** Wen-Jie Fan, Meng-Xi Zhou, Di-Di Wang, Xin-Xin Jiang, Hao Ding

**Affiliations:** 1 The First Affiliated Hospital of Anhui Medical University, Department of Radiology, Hefei, Anhui Province, China.; 2 The First Affiliated Hospital of Anhui Medical University, Department of Gastroenterology, Hefei, Anhui Province, China.; 3 The First Clinical Medical College of Anhui Medical University, Hefei, Anhui Province, China.

**Keywords:** Hepatocellular carcinoma, TMEM147, diagnosis, prognosis, biomarkers

## Abstract

Hepatocellular carcinoma (HCC) is the most common type of liver malignancy with high incidence and poor prognosis. Transmembrane protein 147 (TMEM147) has been implicated in the development of colon cancer. However, the role of TMEM147 in HCC remains unclear. In this study, data of 371 HCC tissues, 50 adjacent nontumor tissues, and 110 normal liver tissues were retrieved from the TCGA and GTEx databases. TMEM147 expression was found to be increased in HCC tissues. High expression of TMEM147 was related to poor prognosis, and TMEM147 was confirmed to be an independent prognostic factor for HCC patients. A receiver operating characteristics (ROC) analysis was performed and showed that the diagnostic efficacy of TMEM147 was significantly higher than that of AFP (0.908 versus 0.746, *p* < 0.001). Furthermore, TMEM147 promoted tumor immune infiltration, and macrophages were the immune cells that predominantly expressed TMEM147 in HCC. Further analysis revealed that TMEM147 mainly impacted the ribosome pathway, and CTCF, MLLT1, TGIF2, ZNF146, and ZNF580 were predicted to be the upstream transcription factors for TMEM147 in HCC. These results suggest that TMEM147 serves as a promising biomarker for diagnosis and prognosis and may potentially become a therapeutic target for HCC.

## Introduction

Hepatocellular carcinoma (HCC), the most common form of liver cancer in adults, ranks 6th in cancer incidence worldwide and causes more than 830,000 annual deaths recorded globally in recent years (Sung et al., 2021). More than 80% of patients with HCC are unresectable at diagnosis, and there are very limited therapeutic options for these patients currently (Iwagami et al., 2016). The medium survival time of patients with HCC is approximately 11 months, with an overall ratio of mortality to incidence of 0.95, indicating an extremely poor prognosis (Simon et al., 2018). Therefore, it becomes an urgent mandate to elucidate the underlying mechanisms and to identify novel biomarkers and molecular therapeutic targets, to improve the diagnosis and treatment of HCC.

Transmembrane protein 147 (TMEM147) is a 25-kDa 7-transmembrane protein that localizes to endoplasmic reticulum (ER) membranes with its N-terminus residing in the lumen and its C-terminus facing the cytosolic side of the membrane (Christodoulou et al., 2020). The human TMEM147 gene is located on chromosome 19q13.12, and its encoded protein is highly conserved in metazoans but is ubiquitously expressed across many human tissues (Dettmer et al., 2010; Christodoulou *et al.*, 2020). It is known that TMEM147 exerts a regulatory role in a wide variety of pathophysiological processes including cell proliferation, apoptosis, and signal transduction regulation (Li et al., 2016). TMEM147 is a core component of the Nicalin-NOMO complex, which modulates embryonic Nodal signaling in vertebrates (Dettmer *et al.*, 2010). TMEM147 inhibits trafficking of the M3 muscarinic acetylcholine receptor to the cell surface (Rosemond et al., 2011). TMEM147 has also been shown to act as a scaffold protein and to mediate NF-κB activation in chondrocytes (Ota et al., 2020). However, the role of TMEM147 in HCC remains hitherto undetermined. In this study, we investigated the clinical significance and diagnostic value of TMEM147, the correlation with immune infiltration, and its regulatory network in HCC, aiming to provide novel insights and potential molecular targets for the diagnosis and treatment of HCC.

## Material and Methods

### Gene expression data collection and processing

Human liver gene expression data from 371 HCC samples and 50 adjacent normal liver samples were obtained from the TCGA database (https://tcga-data.nci.nih.gov/tcga/). To increase the sample size and strengthen the comparative analysis, RNA-seq data from 110 normal liver samples were obtained from the GTEx database (www.gtexportal.org). TCGA Data portal (https://tcga-data.nci.nih.gov/tcga/) and UCSC Xena platform (http://xena.ucsc.edu/) were used to extract the RNA-seq and clinical data. All transcript expression values were normalized to transcripts per million. Genes with a cut-off of 1.0 in |log_2_ fold change| and an adjusted *p*-value < 0.05 were considered differentially expressed genes (DEGs). Data analysis and plot generation were performed using R version 3.4.1, with R packages “ggplot2”, “corrplot”, “pROC”, and “dplyr”. As all data used in this study were publicly available, no additional ethics approval or informed consent was required.

### Tumor Immune Estimation Resource (TIMER)

The TIMER2.0 database (http://timer.comp-genomics.org/) is a publicly available and regularly updated online resource that systematically analyzes gene expression and immune infiltrates across diverse cancer types. In this study, the TIMER2.0 database was used to determine the transcript expression of TMEM147 in different cancer types and to analyze the association between immune infiltration levels and the prognostic value of macrophage infiltration.

### UALCAN

The UALCAN database (http://ualcan.path.uab.edu) is an effective web resource for online analysis and mining of cancer OMICS data using 31 types of cancer RNA-seq and clinical information from the TCGA project (Li et al., 2020). We used this online database to explore the relative expressions of target genes between HCC and normal tissues, as well as in different tumor subgroups based on individual tumor stage, tumor grade, nodal metastasis status, and other clinical pathological features.

### Survival analysis

The Kaplan-Meier Plotter database (http://kmplot.com/analysis/) is an online database containing gene expression data and survival information of over 25,000 samples from 21 tumor types, and sources for the database include GEO, EGA, and TCGA. In this study, the mRNA expression levels of TMEM147 in HCC were divided into high-expression group and low-expression group by the web, and overall survival was estimated by calculating log-rank *p*-value and hazard ratio with 95% confidence interval.

### Correlation analysis

Immuno-modulators associated with TMEM147 were retrieved from the TISIDB database (http://cis.hku.hk/TISIDB/), an integrated repository web portal for tumor-immune system interactions. Gene co-expression correlation between TMEM147 and immuno-modulators was analyzed using Spearman’s correlation test. Data analysis and plot generation were performed using R version 3.6.3, with R packages “ggplot2” and “corrplot”.

### Receiver operating characteristic (ROC) analysis

The ROC analysis was performed using the Wilson/Brown method for TCGA HCC and adjacent normal liver tissues and GTEx normal liver tissues. ROC curve and the area under the curve (AUC) of ROC were analyzed using R package “pROC”. DeLong’s test was adopted to compare the differences in AUC values.

### GO and KEGG analyses

GO and KEGG pathway enrichment analyses were performed using the R package “clusterProfiler”. The most statistically enriched GO terms and KEGG pathways were visualized using the “ggplot2” R package. GO term enrichment analysis includes molecular function (MF), biological process (BP), and cellular component (CC) categories. *P* < 0.05 was set as the cutoff for significant GO and KEGG enrichment analyses.

### Protein-protein interaction (PPI) network analysis

We used the STRING online database (https://string-db.org/) to construct the PPI network. PPIs were obtained with a medium confidence score of 0.4. False discovery rate <0.0005 was set as the threshold for this analysis.

### Construction of transcriptional regulatory network

NetworkAnalyst (http://www.networkanalyst.ca/faces/home.xhtml) is a visual analytics platform specialized in transcriptome profiling, network analysis, and meta-analysis of gene expression data (Zhou et al., 2019). In this study, the transcription factors for TMEM147 were predicted using this online tool, and a transcriptional regulatory network was constructed and visualized by the Cytoscape software V3.5.1.

### Statistical analyses

Data were expressed as mean ± standard deviation. Two-tailed independent sample t-test was used for comparison between the two groups. One-way ANOVA was used for multiple group comparisons. Kaplan-Meier method was used for survival analysis with a log-rank Mantel-Cox test. ROC curves were used to analyze the diagnostic performance of TMEM147 in HCC, and the differences were compared by the DeLong test. Correlations were determined by Spearman’s analysis. Univariate and multivariable survival analyses were performed with Cox proportional hazards model. Differences with *p* < 0.05 were considered statistically significant.

## Results

### Enhanced expression of TMEM147 in HCC

**Figure 1 -:**
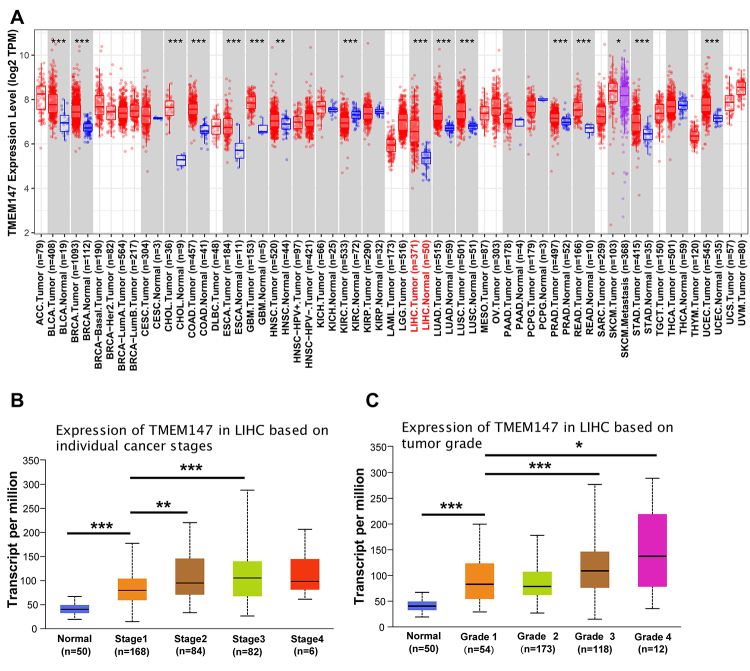
TMEM147 is upregulated in HCC. (A) TMEM147 gene expression in different cancer types. (B-C) TMEM147 gene expression in normal tissues and HCC samples based on individual cancer stages and tumor grade. ^*^ *p* < 0.05, ^**^ *p* < 0.01, ^***^ *p* < 0.001.

The difference in the level of TMEM147 mRNA expression between various cancer tissues and normal tissues was assessed using TIMER based on TCGA data (Figure 1A). TMEM147 was widely expressed in multiple types of cancer and was overexpressed in the vast majority of cancer types including HCC. We further explored the expression of TMEM147 in different subgroups stratified by tumor stage, tumor grade, nodal metastasis status, and other clinical pathological features. It was found that the TMEM147 mRNA level was positively correlated with individual cancer stages and tumor grade (Figure 1B and 1C). However, there was no significant correlation between the TMEM147 mRNA level and nodal metastasis status, patient’s race, patient’s gender, or patient’s age (Figure S1).

### TMEM147 is an independent prognostic factor for HCC

**Figure 2 -:**
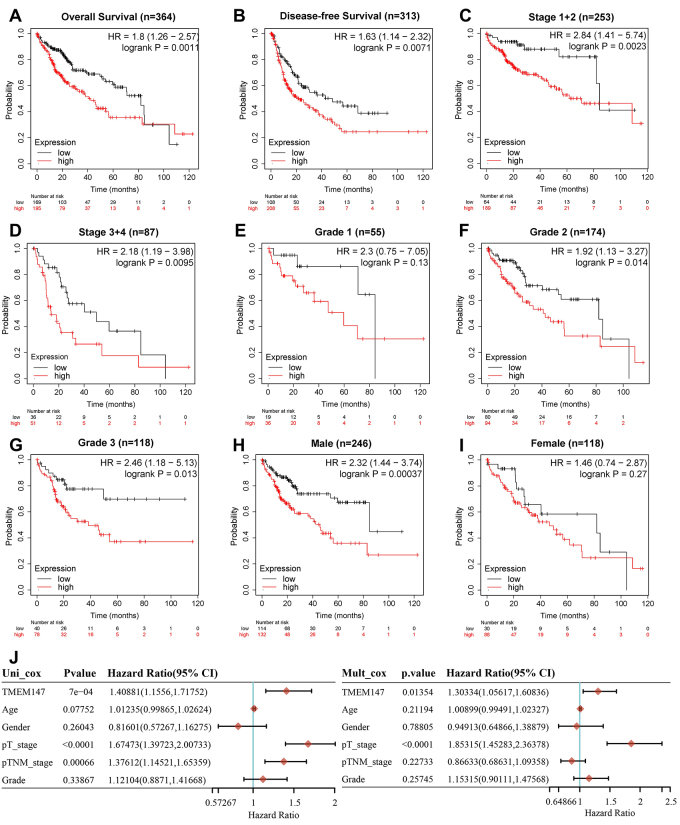
TMEM147 is a prognostic biomarker for HCC. (A-B) Kaplan-Meier curves showing the overall survival and disease-free survival in HCC patients with high or low TMEM147 expression. (C-I) Kaplan-Meier curves showing the overall survival of HCC patient subgroups divided by individual cancer stage, tumor grade, and patient’s gender. (J) Independent prognostic factors determined by univariate and multivariate Cox regression analyses

We evaluated the clinical significance of TMEM147 expression in the prognosis of HCC by using the Kaplan-Meier Plotter database. The results showed that the overall survival and disease-free survival were significantly decreased in TMEM147 high-expression group (Figure 2A and 2B). HCC patients were further subdivided according to individual cancer stage, tumor grade, and patient’s gender, then the prognostic value of TMEM147 in these subgroups was analyzed. It was found that the survival probability of TMEM147 high-expression group was lower in both low- and high-stage HCC patients (Figure 2C and 2D). No significant difference was found between the low and high TMEM147 expression groups in patients with low tumor grade (grade 1) (Figure 2E), while TMEM147 mRNA expression exhibited a significant prognostic value in patients with high tumor grade (grade 2 and grade 3) (Figure 2F and 2G). Interestingly, high TMEM147 expression was significantly associated with worse prognosis in male but not female HCC patients (Figure 2H and 2I). Furthermore, univariate and multivariate analyses were performed to identify survival predictors, which confirmed that TMEM147 was an independent prognostic factor for survival in HCC patients (Figure 2J).

### TMEM147 is a novel diagnostic biomarker for HCC

**Figure 3 -:**
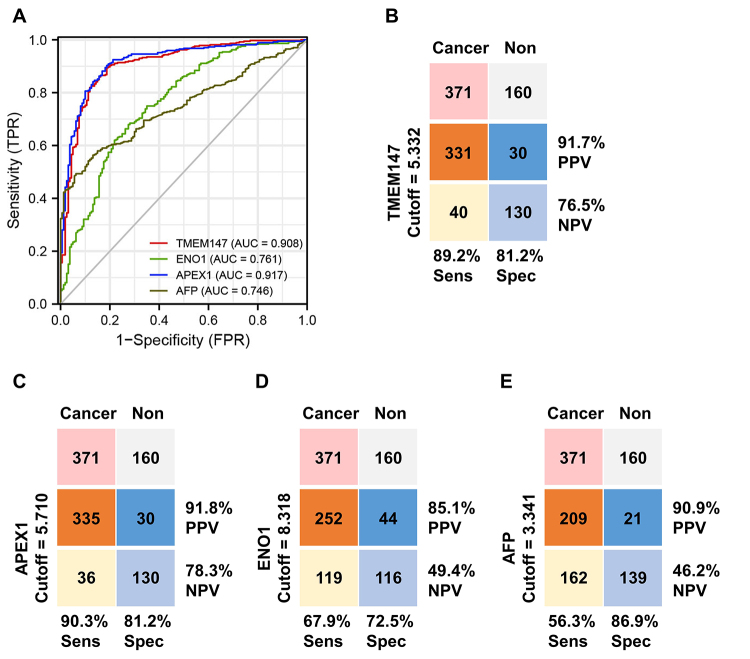
TMEM147 is a diagnostic biomarker for HCC. (A) ROC curve analysis comparing the diagnostic value of TMEM147, ENO1, APEX1, and AFP in HCC patients. (B-E) The positive predictive value (PPV), negative predictive value (NPV), sensitivity (Sens), and specificity (Spec) for TMEM147, ENO1, APEX1, and AFP in HCC patients.

ROC curve analysis was performed to assess the diagnostic value of TMEM147 in distinguishing HCC tissues from non-cancerous tissues. AFP is a widely used diagnostic marker for HCC. APEX1 and ENO1 were recently reported to be novel diagnostic biomarkers for HCC (Zhu et al., 2018; Cao et al., 2020). We found that the AUC value of TMEM147 was significantly higher than that of AFP (*p* < 0.001) and ENO1 (*p* < 0.001), but was comparable to that of APEX1 (*p* = 0.462) (Figure 3A). As shown in Figure 3B to 3E, the positive predictive value (PPV), negative predictive value (NPV), sensitivity (Sens), and specificity (Spec) of TMEM147 were all statistically comparable to those of APEX1 (PPV: *p* = 0.964, NPV: *p* = 0.686, Sens: *p* = 0.628, Spec: *p* = 1.000). Compared with ENO1, the PPV, NPV, and sensitivity of TMEM147 were all significantly higher (PPV: *p* < 0.01, NPV: *p* < 0.001, Sens: *p* < 0.001), but the specificity was comparable (*p* = 0.063). Compared with AFP, the NPV and sensitivity of TMEM147 were significantly higher (NPV: *p* < 0.001, Sens: *p* < 0.001), but the PPV and specificity were comparable (PPV: *p* = 0.729, Spec: *p* = 0.169). Collectively, these results demonstrated that TMEM147 was a reliable diagnostic biomarker for HCC.

### High TMEM147 expression promotes immune infiltration in HCC

**Figure 4 -:**
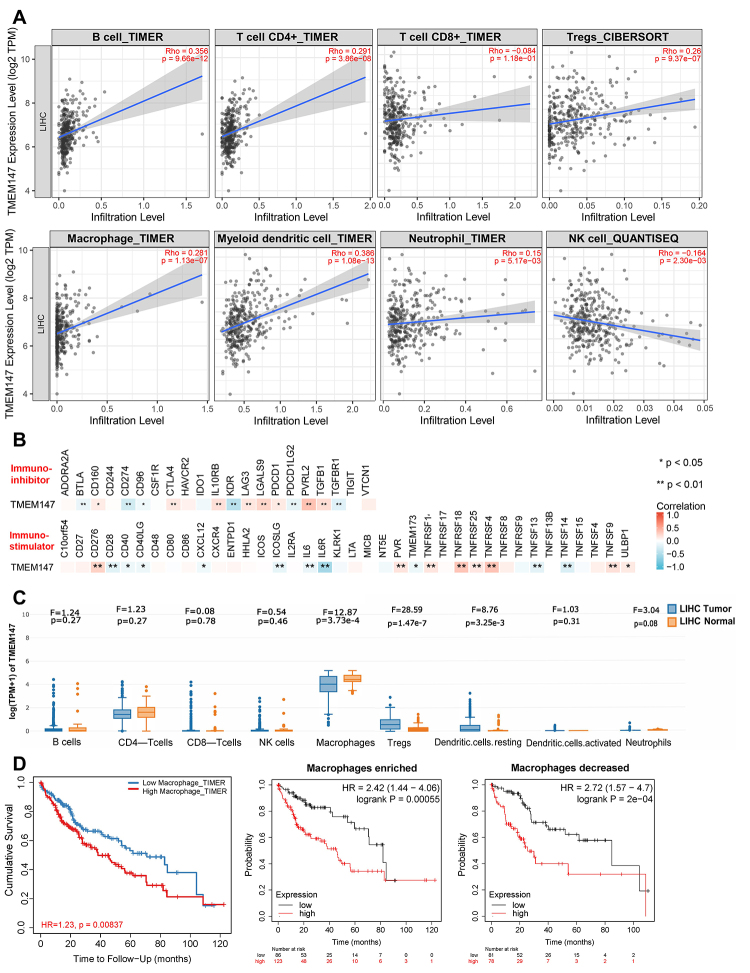
TMEM147 is related to immune infiltration in HCC. (A) Correlation analysis between TMEM147 gene expression and infiltration of B cells, CD4+ T cells, CD8+ T cells, Tregs, macrophages, myeloid dendritic cells, neutrophils, and NK cells. (B) Correlation analysis of gene expression levels between TMEM147 and immuno-modulators. (C) Distribution of TMEM147 expression among different immune cell types in HCC. (D) Kaplan-Meier curves showing the overall survival of HCC patients with high or low macrophage infiltration and HCC patient subgroups divided by macrophage enrichment.

Immune cell infiltration is an important indicator of cancer development (Chen et al., 2019). Its components and activation state reflect tumor biology and predict prognosis (Wang et al., 2020). We further explored the effect of TMEM147 on immune infiltration in HCC by using the TIMER2.0 database. The infiltration levels of B cells, CD4^+^ T cells, T regulatory cells, macrophages, myeloid dendritic cells, neutrophils, and NK cells were significantly correlated with TMEM147 mRNA expression in liver cancer tissues, and no correlation was observed between the infiltration levels of CD8^+^ T cells and TMEM147 expression (Figure 4A). Moreover, the gene expressions of immuno-inhibitors (BTLA, CD160, CD274, etc.) and immuno-stimulators (CD276, CC28, CD40, etc.) exhibited significant correlations with TMEM147 mRNA expression (Figure 4B). These data demonstrated that TMEM147 facilitated immune infiltration in HCC, which was probably attributed to the regulation of the aforementioned immunomodulatory genes. Next, we explored the expression profile of TMEM147 in immune cells. It was found that TMEM147 was mainly expressed in macrophages, and its expression in macrophages was lower in liver cancer tissues compared with the adjacent normal tissues (Figure 4C). High macrophage infiltration predicted poor survival outcome in HCC patients (Figure 4D). TMEM147 exhibited significant prognostic value both in HCC patients with enriched macrophage infiltration and with decreased macrophage infiltration (Figure 4D). These results indicated that TMEM147 significantly affected the immune activity of HCC.

### TMEM147 impacts ribosome signaling in HCC

**Figure 5 -:**
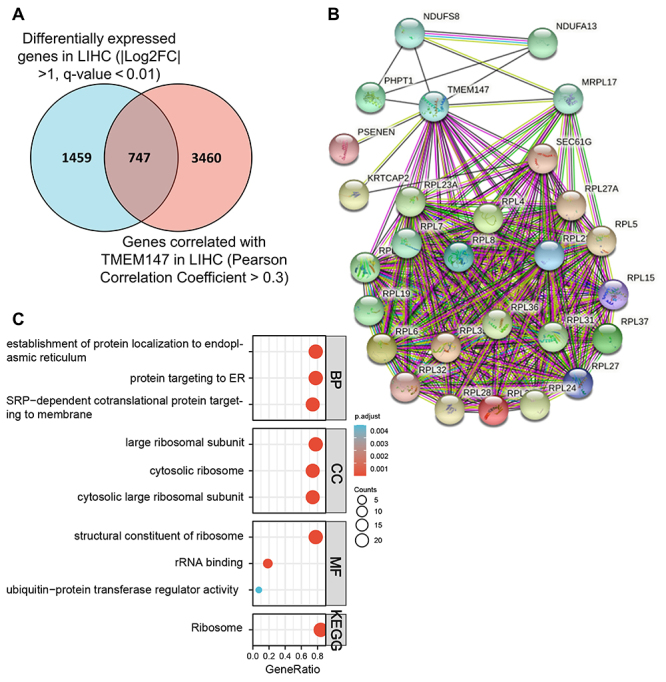
Molecular mechanisms of TMEM147 action in HCC. (A) Venn diagram depicting the common genes between the differentially expressed genes and genes correlated with TMEM147 in TCGA-LIHC dataset. (B) Protein interaction analysis of the filtered common genes using the string database. (C) GO and KEGG pathway enrichment analyses. BP: biological process; CC: cellular component; MF: molecular function.

To explore the potential mechanisms of TMEM147 action in HCC, the common genes between the abnormally expressed genes in liver hepatocellular carcinoma (LIHC) TCGA filtered by GEPIA, and the genes correlated with TMEM147 in LIHC TCGA filtered by UALCAN were analyzed, and a total of 747 common DEGs were screened out (Figure 5A). Protein interaction analysis was further conducted using the String database. A total of 27 proteins were identified to have interactions with TMEM147 (Figure 5B), and these genes were further subjected to GO and KEGG pathway enrichment analyses. Establishment of protein localization to the endoplasmic reticulum, protein targeting to ER, and SRP-dependent cotranslational protein targeting to membrane were the most enriched biological processes. Large ribosomal subunits, cytosolic ribosomes, and cytosolic large ribosomal subunits were the top enriched GO terms in cellular component. Structural constituents of ribosome, rRNA binding, and ubiquitin protein transferase regulator activity were the top enriched molecular functions. KEGG pathway analysis revealed that only one pathway, namely, the ribosome pathway, was enriched significantly (Figure 5C). Taken together, these results indicated that TMEM147 might regulate HCC progression by affecting ribosome signaling.

### Transcription factor analysis for TMEM147 in HCC

**Figure 6 -:**
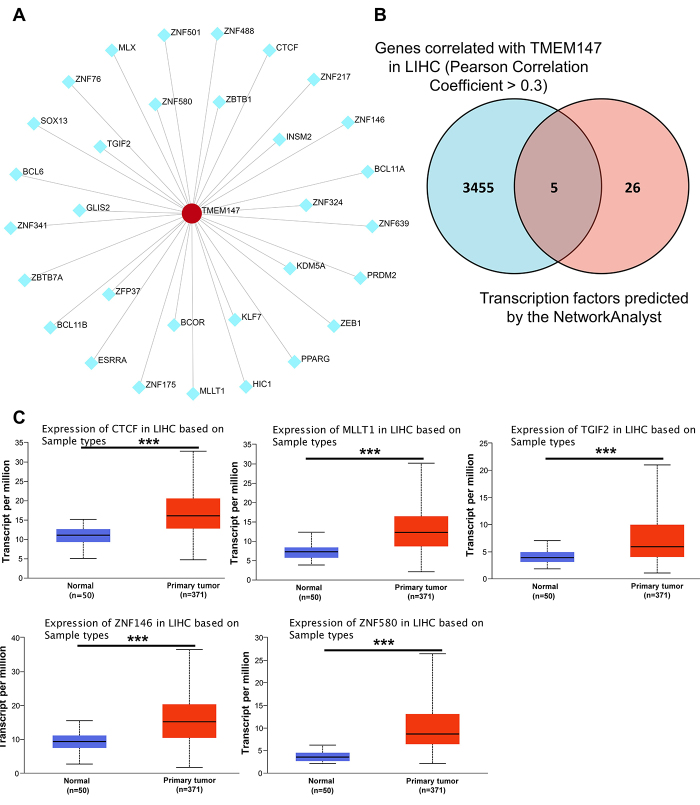
Transcription factor analyses. (A) Transcriptional regulatory network of TMEM147. (B) Venn diagram depicting the overlap between the predicted transcription factors and genes correlated with TMEM147 in TCGA-LIHC dataset. (C) Gene expression analyses of CTCF, MLLT1, TGIF2, ZNF146, and ZNF580 in HCC based on UALCAN database. ^***^ *p* < 0.001.

We further constructed a transcriptional regulatory network using the NetworkAnalyst online platform. It was found that 31 transcription factors can regulate the expression of TMEM147 (Figure 6A). Among these transcription factors, CTCF, MLLT1, TGIF2, ZNF146, and ZNF580 were significantly co-expressed with TMEM147 in LIHC TCGA (Figure 6B). Moreover, the gene expression levels of these 5 transcription factors were found to be significantly upregulated in HCC (Figure 6C). These results suggested a possible explanation for TMEM147 upregulation in HCC.

## Discussion

Transmembrane (TMEM) protein family is a group of proteins that spans completely or partially through biological membranes such as cytoplasmic, mitochondrial, ER, and Golgi membranes (Schmit and Michiels, 2018). Many of TMEM family members are poorly described, and a detailed understanding of their structure, functions, and mechanisms is still lacking. Available evidence suggests that aberrant expression of TMEM family proteins is implicated in the pathogenesis of cancer. Some of TMEM proteins act as tumor suppressors such as TMEM7, TMEM25, TMEM97, and TMEM176A; while a large number of them act as oncogenes such as TMEM14A and TMEM158 (Schmit and Michiels, 2018). TMEM147 was firstly described by Dettmer *et al.* (2020) and was found to be a core component of the nicalin-NOMO complex which localizes to the ER. Subsequent studies revealed that TMEM147 regulates the M₃ muscarinic acetylcholine receptor function, mediates NF-κB activation, interacts with lamin B receptor and affects cholesterol homeostasis, and regulates ER composition and extent (Rosemond et al., 2011; Christodoulou et al., 2020; Ota et al., 2020; Maimaris et al., 2021). However, research concerning its function in cancer is extremely limited. Therefore far, to our knowledge, only one study by Feng *et al*. reported that TMEM147 expression is significantly increased in colon cancer tissues and is identified as a hub gene in colon cancer (Feng *et al.*, 2019). In this study, we firstly demonstrated that TMEM147 was overexpressed in HCC and positively correlated with cancer stage and tumor grade. High expression of TMEM147 predicted poor prognosis and was an independent prognostic factor for HCC patients. Furthermore, the sensitivity and negative predictive value of TMEM147 were significantly higher than AFP, which is currently considered the gold standard noninvasive diagnostic marker for HCC. These results clearly indicate that TMEM147 is a reliable novel biomarker for the diagnosis and prognosis of HCC.

Immune infiltration is widely recognized as an important hallmark of tumors (Jiang et al., 2018). As an essential component of the tumor microenvironment, infiltrating immune cells can alter the immune status of the tumor, and patterns of infiltrating immune cell types have been linked to tumor progression and predict prognosis (Meng et al., 2019; Stewart et al., 2019). In this study, we demonstrated that high expression of TMEM147 was often accompanied by infiltration of various types of immune cells, and the expression patterns of TMEM147 were closely associated with that of immuno-modulators in HCC tissues. In addition, we also revealed that TMEM147 was predominantly expressed in macrophages. High levels of TMEM147 predicted poor prognosis, whether in HCC patients with enriched macrophage infiltration or those with decreased macrophage infiltration.

To understand the regulatory mechanisms upstream and downstream of TMEM147, GO and KEGG pathway enrichment analyses and transcription factor analysis were conducted. It was found that TMEM147 mainly impacted the ribosome signaling pathway and was predicted to be transcriptionally regulated by CTCF, MLLT1, TGIF2, ZNF146, and ZNF580 in HCC. Ribosome is the cellular factory responsible for protein synthesis, and ribosome biogenesis is directly linked to cell proliferation and growth (Ren et al., 2011; Sharif et al., 2019). In this study, of the 27 genes screened, 21 are structural constituents of the 60S ribosomal subunit. Therefore, a reasonable speculation is that TMEM147 promotes tumor progression possibly by affecting protein biosynthesis in HCC. CTCF, MLLT1, and TGIF2 are transcriptional regulators that play crucial roles in the transcription of important proto-oncogenes such as FOXM1, MYC, HoxA gene family, and OCT4 in multiple cancer types including HCC, esophageal cancer, and lung adenocarcinoma (Zhang et al., 2017; Du et al., 2019; Liu et al., 2022). ZNF146 and ZNF580 are both members of the C2H2 family of Zinc finger proteins. ZNF146 was reported to be targeting to distinct sites within LINE-1 sequences at thousands of locations in the genome and to promote tumorigenesis of HCC, gastric cancer, and colorectal cancer (Ma et al., 2020; Bao et al., 2021; Zhu et al., 2021; Creamer et al., 2022). ZNF580 was reported to regulate the gene expression of VEGF-A, IL-8, MMP2, and SMAD2 and is involved in angiogenesis, endothelial inflammation, and cell migration and proliferation (Sun et al., 2010; Stenzel et al., 2018). These 5 proteins were predicted to be transcription factors for TMEM147, indicating a possible mechanism that mediates the transcriptional upregulation of TMEM147 in HCC.

In conclusion, this study analyzed data from TCGA and GTEx databases and demonstrated that TMEM147 is a novel diagnostic and prognostic biomarker for HCC. We also demonstrated that macrophages are the immune cells that predominantly express TMEM147, and high expression of TMEM147 promotes immune infiltration in HCC. To the best of our knowledge, this is the first study to explore the clinical value and underlying molecular mechanisms of TMEM147 in HCC, highlighting the potency of TMEM147 as a diagnostic and prognostic biomarker and a therapeutic target for HCC. However, several limitations in this study should be noted. First, all of the data used in this study were transcriptomic data obtained from public databases. Further studies are required to validate these results by using paired specimens of tumor tissues and adjacent normal tissues. Second, we conducted diagnostic analysis in all HCC patients from TCGA database, thus the diagnostic value of TMEM147 in different tumor stages, especially in early stages, remains undetermined and warrants further study. Finally, *in vitro* and *in vivo* experiments are needed to verify the upstream regulators and downstream signaling pathways identified in this study.

## Supplementary material

The following online material is available for this article:

Figure S1 - TMEM147 gene expression in normal tissues and HCC samples based on nodal metastasis status, patient’s race, patient’s gender, and patient’s age.
