# Supplementary material for: TMEM147 is a novel biomarker for diagnosis and prognosis of hepatocellular carcinoma
Source: Genet Mol Biol. 2023 Jun 16;46(2):e20220323. doi: 10.1590/1678-4685-GMB-2022-0323 (PMC10278923; doi:10.1590/1678-4685-GMB-2022-0323)
Supplement: Figure S1 - [file 1415-4757-GMB-46-02-e20220323-s1.pdf]

Genetics and Molecular Biology

https://doi.org/10.1590/1678-4685-GMB-2022-0323

Supplementary Material to "TMEM147 is a novel biomarker for diagnosis and prognosis of hepatocellular carcinoma"

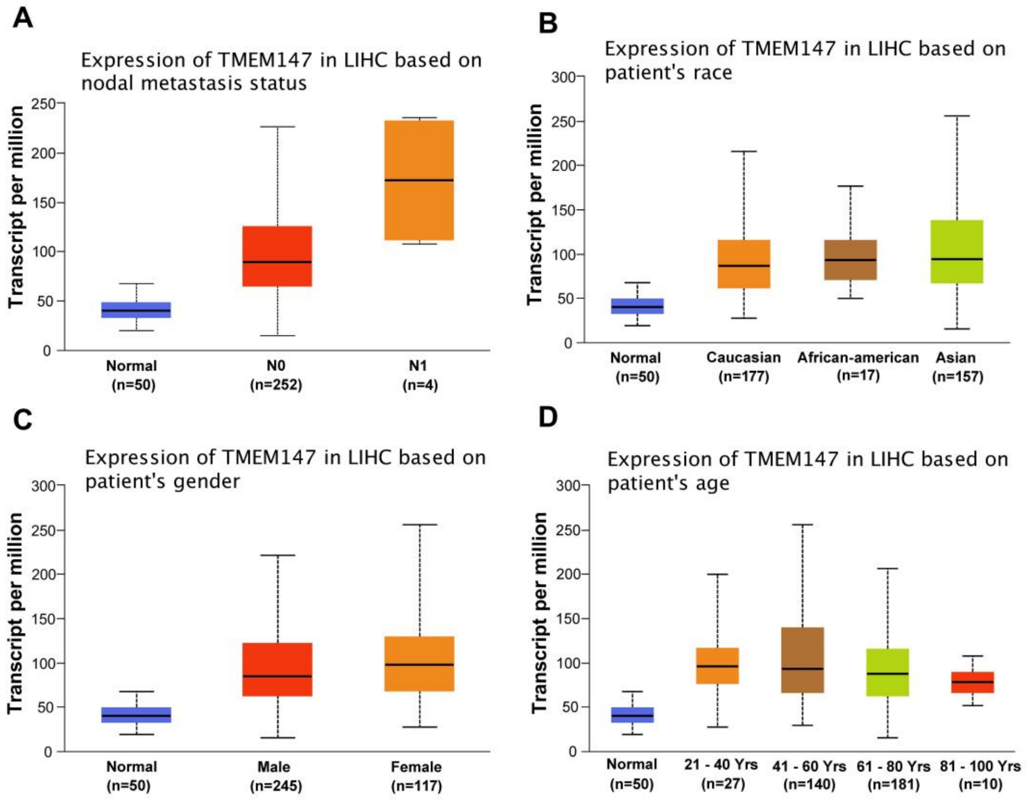

Figure S1 - TMEM147 gene expression in normal tissues and HCC samples based on nodal metastasis status, patient's race, patient's gender, and patient's age.
